# The p38 MAPK Components and Modulators as Biomarkers and Molecular Targets in Cancer

**DOI:** 10.3390/ijms23010370

**Published:** 2021-12-29

**Authors:** Laura García-Hernández, María Belén García-Ortega, Gloria Ruiz-Alcalá, Esmeralda Carrillo, Juan Antonio Marchal, María Ángel García

**Affiliations:** 1 Faculty of Pharmacy, Complutense University of Madrid, 28040 Madrid, Spain; laugarher99@gmail.com; 2 Biopathology and Regenerative Medicine Institute (IBIMER), Centre for Biomedical Research (CIBM), University of Granada, 18100 Granada, Spain; mbgarcia87@hotmail.com (M.B.G.-O.); gloriara1989@hotmail.com (G.R.-A.); esmeral@ugr.es (E.C.); 3 Instituto de Investigacioón Biosanitaria ibs. Granada, University Hospitals of Granada, University of Granada, 18100 Granada, Spain; 4 Excellence Research Unit “Modeling Nature” (MNat), University of Granada, 18100 Granada, Spain; 5 BioFab i3D-Biofabrication and 3D (Bio)Printing Laboratory, University of Granada, 18100 Granada, Spain; 6 Department of Human Anatomy and Embryology, Faculty of Medicine, University of Granada, 18071 Granada, Spain; 7 Department of Biochemistry and Molecular Biology III and Immunology, University of Granada, 18071 Granada, Spain

**Keywords:** kinases, MAPK, p38, cancer, therapy, biomarkers, isoforms

## Abstract

The mitogen-activated protein kinase (MAPK) family is an important bridge in the transduction of extracellular and intracellular signals in different responses at the cellular level. Within this MAPK family, the p38 kinases can be found altered in various diseases, including cancer, where these kinases play a fundamental role, sometimes with antagonistic mechanisms of action, depending on several factors. In fact, this family has an immense number of functionalities, many of them yet to be discovered in terms of regulation and action in different types of cancer, being directly involved in the response to cancer therapies. To date, three main groups of MAPKs have been identified in mammals: the extracellular signal-regulated kinases (ERK), Jun N-terminal kinase (JNK), and the different isoforms of p38 (α, β, γ, δ). In this review, we highlight the mechanism of action of these kinases, taking into account their extensive regulation at the cellular level through various modifications and modulations, including a wide variety of microRNAs. We also analyze the importance of the different isoforms expressed in the different tissues and their possible role as biomarkers and molecular targets. In addition, we include the latest preclinical and clinical trials with different p38-related drugs that are ongoing with hopeful expectations in the present/future of developing precision medicine in cancer.

## 1. Introduction

Currently, a large part of the resources allocated to global health are being directed towards the study and prevention of cancer, as it is one of the main causes of death worldwide [1]. As a result, the race to find new therapies aimed at reducing its prevalence and mortality is one of the great challenges currently facing the scientific community. 

Thanks to these efforts, it has been possible to relate the appearance of various types of cancer with the mutational state of some important genes. By identifying certain molecular patterns that do not function properly, it would be possible to successfully intervene with different therapies aimed at correcting or blocking these alterations involved in the development and survival of tumor cells [2]. Specifically, cancer is usually correlated with the acquisition of mutations that alter key cell signaling pathways, and mitogen-activated protein kinases (MAPKs) have been identified as a family of kinases that are highly implicated in different cancer processes [3].

In 1991, the sequences of the first MAPKs studied in mammals (ERK1, ERK2, and ERK3) were revealed, identifying them as member enzymes of this newly discovered specific family of protein kinases [4]. MAPKs are regulated by phosphorylation cascades, as they are activated by the action of a mitogen-activated protein kinase kinase (MAPKK), which phosphorylate a MAPK. This MAPKK, in turn, is activated when it is phosphorylated by another kinase targeting it (MAPKKK). In summary, two serially activated protein kinases (MAPKKK and MAPKK) result in the activation of a MAPK, although additional kinases may also be required to induce this three-kinase module [3,5]. 

**Figure 1:**
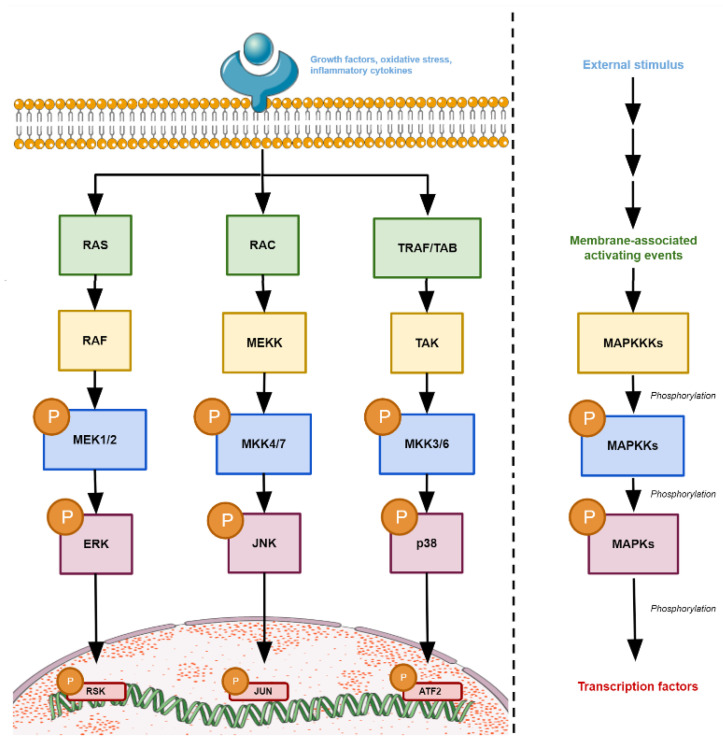
Scheme of the different MAPK family’s phosphorylation system. The modules shown are representative of the different connections between the components of the pathways that can occur for the respective MAPK phosphorylation systems. There are multiple components that act as external stimuli and are able to produce different events at the plasma membrane, mediated by activators belonging to the Ras/Rho GTPase family, such as RAS or RAC, or by the recruitment of adaptor proteins such as TRAF. These activate the MAPK cascade with their respective MAPKKKK, MAPKKK, and MAPKK components. Figure created using Servier Medical Art by Servier, which is licensed under a Creative Commons Attribution 3.0 Unported License: https://smart.servier.com (accessed on 23 November 2021).

MAPK signal transduction pathways are ubiquitous and highly conserved regulatory mechanisms in eukaryotic cells which promote coordinated and integrated responses to numerous stimuli through protein kinase-type receptors, leading to important cellular physiological effects. MAPKs phosphorylate target substrates at Ser or Thr residues, followed by a Pro residue, suggesting limited specificity [6]. This specificity is also mediated by specific MAPKK docking sites for MAPKs, as well as scaffolding proteins, distinct polypeptides that function by sequestering components of the MAPK pathway to maintain pathway integrity and signaling efficiency [7]. Finally, MAPKs are inactivated by both generic phosphatases and dual-specificity phosphatases [8]. In mammals, 14 MAPKs have been identified and grouped into three main groups: extracellular signal-regulated kinases (ERK), Jun N-terminal kinases (JNK) and p38 isoforms (α, β, γ, δ) [7,8,9] (Figure 1).

In recent years, the cross-interaction of the JNK and p38 families has been studied in greater depth, elucidating their dual role in different types of cancer, depending in many cases on the cellular context and the specific individual [10]. Both the Jun N-terminal kinase (JNK) and p38 kinase pathways are de-named, stress-activated protein kinase pathways (SAPKs) and are often dysregulated in cancer [11,12]. Most environmental factors, as well as different types of genotoxic stress, sensitize these kinases, implicating them in a wide cellular response that leads to inflammatory processes that control the homeostasis of the organism, cell proliferation, differentiation and migration, and a specific response depending on the cell type affected, leading to survival [13]. Upstream activators and downstream targets can overlap between the different MAPK subfamilies, allowing cross-interaction and feedback signaling reactions between MAPK family components [14].

In this review, we highlight the broad functionalities of the different components of the p38 MAPK molecular signaling pathway, considering the role of even the less known p38 isoforms, in the development of numerous cancer-related processes, highlighting their dual role in multiple tumor types and their great viability as cancer biomarkers or potential therapeutic agents.

## 2. The p38 MAPK Pathway

Four genes with high sequence homology have been identified that code for MAPK/p38: *MAPK14*, *MAPK11*, *MAPK12*, and *MAPK13*, which code for p38α, p38β, p38γ, and p38δ, respectively [15], with p38α being 75% identical to p38β and p38γ being 75% identical to p38δ [16]. p38α/*MAPK14* is expressed in almost all cellular types, whereas the other isoforms are expressed in specific tissues. For example, p38β is mainly found in the brain, p38γ is expressed in skeletal muscles, and p38δ is observed in kidneys, the pancreas, the small intestine, and testis [12,14]. It has been shown that p38α and p38β are related proteins that may share functions, although p38β is normally less expressed in cell types. On the other hand, p38γ and p38δ are more restricted in their expression, therefore it is possible that they have specialized functions [17]. In general, p38α and p38β act together in various processes of a very different nature, such as sex determination, cardiac development, inhibition of mitotic entry, and the induction of immune T cells [18]. It has been shown that, in certain cell lines, p38γ and p38δ can carry out the same functions in processes of tissue regeneration and immune responses [19]; however, there is not genetic evidence supporting the notion that p38γ or p38δ realize functions of p38α [16]. It should be noted that the downregulation of p38α sometimes induces the activation of p38γ and/or p38δ [20]. Therefore, the individual and combined role of the four p38 isoforms must be further explored to understand the biological functions of this intricated signaling pathway.

### 2.1. p38 MAPK Activation and Regulation

The great complexity of the p38 MAPK pathway lies in addition to its mechanism of action, in its regulation at various levels by activators and inhibitors of different natures.

In mammals, p38 isoforms are activated in response to different stresses produced in the cellular environment as well as the liberation of inflammatory cytokines such as interleukin-1 (IL-1) and tumor necrosis factor-alpha (TNF-α) in processes of oxidative stress during UV radiation exposition, hypoxic conditions, or during ischemia [13]. 

As with the other MAPK families, the p38 pathway involves the canonical cascade activation of certain small proteins of the Rho GTPase family, such as Rac or Cdc42, which are upstream of the MAPK module discussed above [6]. Some of the characteristic MAPKKKs (or MAP3Ks) that activate p38 are MEKK3/4, TGFβ-activated kinase 1 (TAK1), and apoptosis signal-regulating kinase 1 (ASK1) [13]. The main MAPKKK that activates the p38 pathway in response to oxidative stress, induces proapoptotic signaling through a reduced form of the protein thioredoxin with antioxidant properties (TRX) [21]. In addition, binding of TAK1-binding protein 1 (TAB1) would induce a conformational rearrangement of p38 in its activation loop that would facilitate p38 autophosphorylation [22]. However, these mechanisms have not yet been adequately characterized. Downstream of the pathway are founded the MAPKKs (or MAP2Ks), including MKK3 and MKK6 [23]. Moreover, it has been shown that MKK4 can phosphorylate p38; however, it is also an activator of the JNK pathway, presenting the crosstalk in the signaling of both pathways [24]. MKK3/6 activate p38 by the phosphorylation of both residues Thr180 and Tyr182 in a Thr–Gly–Tyr motif [13]. The biological consequences resulting from p38 activation depend on the magnitude as well as the duration of the signal, therefore the mechanisms of p38 inactivation are crucial for regulating the functionality of this pathway.

On the other hand, there is a noncanonical p38 activation pathway. This occurs, for example, in T cells, where p38 can be activated by T-cell receptor (TCR)-mediated phosphorylation at a Tyr323 residue by the 70-kDa protein kinase ζ-chain-associated protein kinase (ZAP70) [25].

As mentioned before, the p38 module is also bound by scaffolding proteins that play a crucial role in the specificity of its signaling. These proteins do not possess catalytic activity themselves, but regulate the activity of the p38 cascade by promoting the conformational changes necessary for its activation and controlling its cellular localization [26]. 

#### 2.1.1. p38 MAPK Regulation by Phosphatases

p38 activation occurs by dual phosphorylation at Thr180 and Tyr182 residues located in the activation loop, therefore one way to de-phosphorylate these sites is by the actuation of phosphatases at several levels, acting also upstream of dephosphorylate MAP2Ks. Within this group are members of the dual specificity phosphatases (DUSP) family of phosphatases, which includes MAPK phosphatases (MKP). Notably, these phosphatases are activated even by stimuli that activate p38 [27]. DUSP4 is considered an oncological biomarker, although its functionality is not entirely clear. Different studies have been conducted in several types of cancer, showing that DUSP4 has an oncogenic role, however others’ analysis have suggested a tumor suppressor activity [28]. A lower concentration of DUSP4 was observed in breast cancer tissues compared to normal tissues [29], as well as in regions of colorectal cancer [30]. In papillary thyroid carcinoma, higher DUSP4 expression has been shown to correlate with a better clinical outcome [31]. In a study on renal carcinoma, it was shown that loss of DUSP4 expression could lead to a poor prognosis of the cancer [28]. Furthermore, the involvement of DUSP4 expression and chemoresistance has been shown to be positively correlated in gastric and breast cancers [32,33]. Members of the PP2 family of serine/threonine phosphatases have also been found to inactivate p38. For example, PP2C can inactivate p38 indirectly by targeting MAP2Ks or directly on p38 by dephosphorylation of the Thr180 residue [34]. In addition, the PP2A form can interact with the active form of p38 to inactivate it, thus its inhibition would maintain the phosphorylated state of p38. It has been observed in several types of tumor cells and macrophages that PP2A inhibition is associated with an increase in p38 activity. However, the role of PP2A depends on the cell type and stimuli [35]. One study showed that the pharmacological inhibition of PP2A could confer resistance to treatment with the inhibitors of cutaneous squamous cell carcinoma cell proliferation, whereas the overexpression of wild-type PP2A led to the opposite results [36]. In addition, one study identified PP2A as an alternative drug target for cells that have shown resistance to the inhibition of other components of the MAPK pathway in PANC-1 pancreatic cancer cells. A PP2A activator, DT-061, was able to decrease the viability of these cells, along with reducing c-Myc expression [37].

### 2.1.2. p38 MAPK Pathway Regulation by microRNAs 

MicroRNAs (miRNAs) are small, conserved noncoding RNAs that act at the post-transcriptional level, inducing the silencing of complementary mRNA targets once the Argonaute proteins assemble in miRNA-induced silencing complexes (miRISCs) [38]. Most of research has indicated that miRNAs are part of the homeostatic signaling mediated by the p38 pathway in response to numerous activators, playing a dual role in the development of cancer and inflammation. For example, miRNA-17-92 is upregulated in several types of cancer such as breast cancer, colon cancer, and gastric cancer, promoting protumor functions and subsequent cancer progression. Moreover, the miR17-92 cluster also regulates ASK1 [39]. In addition, miR-196a repressed p38-dependent cell migration in response to VEGF by binding to the 3’UTR region of annexin A1 mRNA in esophageal, breast and endometrial cancer [40]. Of note is the role of miR-200 family members together with p38 and oxidative stress in liver cancer cells, such that the p53-dependent expression of miR200a-3p promotes cell death by inhibiting a p38/p53/miR-200 feedback loop [41]. Furthermore, miR-141 and miR-200a directly target p38α regulating the ROS response in ovarian cancer and p38α deficiency by overexpression of miR-141 and miR-200a enhanced tumor growth and sensitized cancer cells to chemotherapeutic treatments [42]. Recently, it has been found that the aberrant expression of miR-139-5p in uterine leiomyoma was associated with an overexpression of collagen type 1 protein and phosphorylated p38 MAPK protein, increasing cell migration [43]. The expression of some molecules involved in the regulation of the immune system can be correlated with MAPK activity. For example, a study suggested that MAPK may regulate PD-L1 expression in liver cancer [44]. PD-L1 inhibition T-cell function and the activation of EGFR by its ligand can induce the positive regulation of this molecule. In this sense, it has been suggested that the EGFR inhibitor Gefitinib was able to decrease PD-L1 expression and inhibition of T-cell-mediated lysis of liver cancer cells through EGFR activation [45]. Thus, anti-PD-L1 antibody therapy could be used to decrease immunosuppression in cancer patients with uncontrolled EGFR activation [46]. EGFR signaling has been shown to provide PD-L1 mRNA with increased stability and half-life, thus proposing the involvement of an epigenetic regulatory mechanism. In addition, the expression levels of miR-675-5p differ in different cancers contributing to several functions such as cell proliferation and invasion, as well as metastasis [47,48]; the downregulation of miR-675-5 by p38 MAPK activation provides stability to the PD-L1 RNA through the 3’-UTR region and thus causes PD-L1 accumulation. For this reason, the EGFR/p38 MAPK axis has been involved in the regulation of PD-L1 through miR-675-5p with interesting potential on the immunotherapy of hepatocellular carcinoma that needs more in-depth studies [45]. 

On the other hand, the p38 MAPK pathway has been also involved in cervical cancer through the miR-409-3p/CDK8 axis [49]. In glial tumors, a modulation of the p38 MAPK signaling pathway through miR-106a-5p suppressed glioma stemness and radioresistance [50] In pancreatic cancer, miR-27a-3p was involved in the induction of apoptosis via the modulation of p38 MAPK through the upregulation of the long noncoding RNA DGR5 [51].

These findings suggest that miRNAs may become the main regulators of the long-term specificity and sensitivity of p38 and other signaling pathways. However, since there are numerous identified miRNAs that regulate the p38 MAPK pathway in different tumor models with different consequences, its use as biomarkers or therapeutic targets is still far from being readily applied in clinical practice.

### 2.2. Substrates and Subcellular Localization of p38 MAPK

MAPK p38 can be found in both the cytoplasm and the nucleus, and its location determines its signaling specificity [52]. p38 is mainly found in the cytoplasm of resting cells, in an inactive, nonphosphorylated form. Once p38 is activated, an important pool remains in the cytoplasm to regulate the functions of some cytoplasmic proteins. However, depending on the age of cells, the cell type, or the inductor stimulus, another phosphorylated p38 pool is found in the nucleus, giving it access to nuclear substrates [53]. For example, the UV radiation enhances the transport of p38 to the nucleus [14]. Conversely, in resting cells, p38 can also be present in the nucleus and be exported to the cytoplasm [53].

In response to inducers of DNA damage, such as radiotherapy or chemotherapy used in cancer treatment, the nuclear localization of p38 depends on its phosphorylation, but does not depend on its own kinase activity [54]. Since p38 does not have a nuclear localization signal (NLS), this kinase is translocated to the nucleus thanks to the associated proteins such as importins or proteins with an NLS sequence. Moreover, p38 is exported to the nucleus in its dephosphorylated form using MK2 without requiring a nuclear export signal (NES) [55].

The p38 substrates include other protein kinases and transcription factors, 55% of which are found in the nucleus [14]. Most of these are DNA- and RNA-binding proteins that regulate gene expression. Among them, one of the first reported p38 substrates, the CHOP transcription factor GADD153, leads to cell cycle arrest between the end of the G1 phase and the beginning of the S phase, once it is activated after ROS-induced DNA damage, allowing DNA reparation [56]. Cyclic AMP-2-dependent transcription factor (ATF2), signal transducer and activator of transcription-3 (STAT-3), and p53 are other transcription factors activated by p38 MAPK leading to cell growth control, facilitating the induction of apoptosis, inducing the immune system modulation, and generating a specific response to DNA damage [14]. In fact, the phosphorylation of p53 by p38 regulates the G2/M transition [57] and the p38 target, and HMG box transcription factor 1 (HBP1) represses cell cycle progression in G1. Recently, it has been shown that the activation of endothelial p38 by IL-1β regulates miR-31 transcription through the activation of c-fos and GATA2 and, in turn, miR-31 inhibits E-selectin expression repressing the trans-endothelial adhesion phenomenon and the migration of colon cancer cells [58]. There are many other transcription factors that are not directly targeted by p38, but are targeted by downstream subtracts of p38 such as MK2 and the substrates Cdc25b and Hur [59]. The transcription factor CREB is also phosphorylated by MK2 and other substrates such as stress-activated protein kinase 1 and 2 (MSK1 and MSK2) [60]. In addition, some proteins can be dually phosphorylated by p38 and MK2 and they may function as mechanisms to favor a more fine-grained regulation, thus preventing the inappropriate activation of effectors [59].

In conclusion, most studies have suggested that p38 regulates the inhibitors or activators of transcription, in addition to chromatin modulation, favoring or not the transcription of many genes implicated in different cellular processes [6].

**Figure 2:**
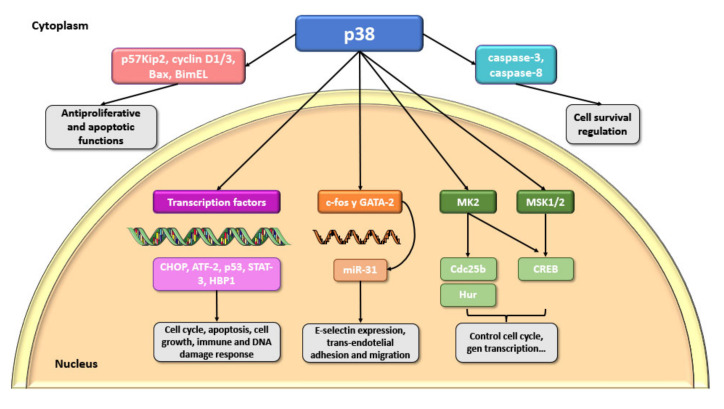
Different substrates and targets of p38 and their related activities. p38 phosphorylates an immense number of proteins and can exert an indirect effect on an even wider network of molecules, highlighting the many functionalities of this pathway. Most of the target substrates are found in the nucleus, although other proteins have also been suggested to be activated by p38 in the cytoplasm. It has been shown that the p38 MAPK pathway can lead to rapid control of processes such as cell cycle progression, DNA damage repair, or mRNA processing. In addition, it phosphorylates many transcription factors involved in gene expression mechanisms, which can lead to cytokine expression and other inflammatory events. In addition, p38 can also activate by phosphorylation a number of protein kinases named as MAPKAPKs, including MK2 and MSK1/2 [61]. Figure created using Servier Medical Art by Servier, which is licensed under a Creative Commons Attribution 3.0 Unported License: https://smart.servier.com (accessed on 23 November 2021).

On the other hand, the cytosolic substrates of p38 include proteins with antiproliferative functions such as p57Kip2 and cyclin D1/3, and apoptotic functions such as Bax and BimEL. Moreover, p38 can regulate cell survival by phosphorylation of caspase-3 and caspase-8 [14] (Figure 2).

## 3. Role of the p38 MAPK Pathway in Cancer

There is strong evidence for a role of p38α as a tumor suppressor, mainly mediated by the negative regulation of the cell cycle and the activation of apoptosis. Moreover, the induction of terminal differentiation is involved in its role as a tumor suppressor [62]. However, it has been shown that this isoform may also have oncogenic activities, mediated by its implication in the development of important processes of cancer development and prognosis, such as invasion, proliferation, angiogenesis, and inflammation [10]. On the other hand, p38γ and p38δ isoforms have been recently shown to play a crucial role in several diseases, including cancer [63], although the role of p38β is not yet fully known [64].

### 3.1. Proliferation, Survival, and Differentiation

As we know, uncontrolled proliferation is a characteristic process in cancer, and the p38 MAPK pathway plays a key role in regulating cell cycle progression at different points by mechanisms that can depend on transcription machinery. In addition, it can modulate antagonic cellular programs for cell survival, differentiation, or cell death induction, resulting in significant effects on the progression of several types of cancers [10].

As discussed before, p38α can negatively regulate cell cycle progression at the G1/S and G2/M transitions by different processes, such as the downregulation of cyclins and the upregulation of cyclin-dependent kinase (CDK) inhibitors, as well as the modulation of the tumor suppressor p53 [57]. p38 MAPKs may also trigger premature senescence in primary cells, which is an arrest of ongoing proliferative activity by oncogenes such as *HRAS*, functioning as an anti-tumor defense mechanism by inducing p53 phosphorylation [65]. This function has been observed in a highly conserved manner in the p38α isoform in several cell types such as hepatocytes, cardiomyocytes, fibroblasts, lung and hematopoietic cells [10]. This effect of p38α may be mediated by the JNK pathway by a negative regulation of the epidermal growth factor receptor (EGFR), depending on the cell type [66]. However, it has been shown that p38α can also be associated with the positive regulation of proliferation, for example, in hematopoietic cells and in several cancer cell lines [10]. These opposing effects are likely due to the wide variety of kinase activity levels at which p38 acts, together with cross-interactions with other signaling pathways. Moreover, p38α is involved in the induction of apoptosis, which may be mediated by transcriptional or post-transcriptional processes that may affect some death receptors that mediate survival signals or the Bcl-2 proteins that regulate the apoptosis phenomenon. ROS can also activate these apoptotic stimuli through p38α, and it is possible that this mechanism is important for the suppression of tumor initiation, as it can trigger apoptosis in response to the activation of ROS-producing oncogenes in already immortalized cells [67]. It has been proposed that the isoform p38β is able to inhibit the apoptosis in several cell lines and could antagonize the proapoptotic effect of p38α activation.

A quiescent state known as cancer dormancy, induced by p38α through inflammatory and antiapoptotic signals, such as the liberation of IL-6 cytokine, has been also suggested as a mechanism involved in drug resistance. This different activity of p38α could be relevant for tumor suppression, leading to more differentiated and less transformed cells in cancer cell lines, such as in renal carcinoma or colon cancer [10]. 

Ultimately, it is likely that the different effects of p38α pathway activation depends on cell type differences, along with the duration, quality, and intensity of the stimulus and its communication and interaction with other signaling pathways.

On the other hand, it has been shown that p38β could be involved in cell proliferation, thanks to its relationship with integrin-αv, as it maintains cell proliferation in keratinocytes by controlling the translation of c-Myc FAK, p38β, and p90RSK1. Therefore, it has been shown that either chemical inhibition or genetic interference of p38β *MAPK11* in keratinocytes could promote a decrease in c-Myc expression [68].

The analysis of the expression of p38γ and p38δ in both cell lines and patient samples indicate that they have pro- and antioncogenic roles in tumor development and outcome [63]. For example, p38δ has been shown to be silenced in malignant melanoma and cutaneous squamous cell carcinoma of the esophagus according to its tumor suppressor role, whereby its expression is eliminated by a mechanism of methylation modification and transcriptional downregulation of its *MAPK13* gene [69,70].

### 3.2. Metastasis in Relationship with Migration and Inflammation

Chronic inflammation plays a key role in cancer development by promoting cancer survival, along with angiogenesis and cell invasion and metastasis. Studies have shown that these processes are controlled by the p38 MAPK pathway, as that these enzymes regulate the activity and expression of some inflammatory mediators such as different proteases and cytokines that contribute to cancer development and progression [71].

**Figure 3:**
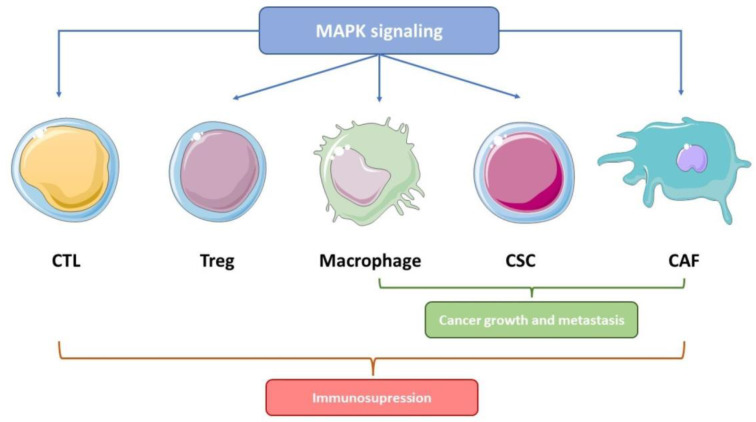
Interactions between MAPK signaling with cells in the tumor microenvironment (TME). The p38 MAPK pathway plays a key role in positive interactions between MAPK signaling with tumor-promoting cells within the TME, including cancer-associated fibroblasts (CAFs), cancer stem cells (CSCs), tumor-associated macrophages (TAMs), and regulatory T cells (Tregs), which, together with the exchange of signals with antitumor cytotoxic T lymphocytes (CTLs), provide an immunosuppressive system for cancer progression. MAPK interactions with CAF, CSCs, and TAMs also promote tumor growth and metastasis. Figure created using Servier Medical Art by Servier, which is licensed under a Creative Commons Attribution 3.0 Unported License: https://smart.servier.com (accessed on 23 November 2021).

The tumor microenvironment (TME) is the collection of cells (normal and immune), molecules (such as cytokines and chemokines), and blood vessels that surround and feed a tumor. In addition, the stroma, which dynamically supports the TME, is formed by the extracellular matrix, a different type of cells, including fibroblasts, infiltrated immune cells, and histological structures such as the blood vessels or connective tissue [72] (Figure 3).

It has been shown that p38α regulates the activation of cyclooxygenase 2 (COX2), which has a proinflammatory activity that has significant effects on cancer progression, such as nonmelanoma skin cancer, breast cancer, or glioma [73,74,75]. In addition, this isoform is involved in the production of cytokines such as TNFα, IL-1, and IL-6, with pro-inflammatory, pro-survival, and angiogenic effects [76]. This can occur at the level of the modulation of transcription factors such as NF-κB [77] or post-transcriptionally by regulating mRNA stability and protein translocation, mainly mediated by MK2 kinase [53]. However, p38β does not appear to mediate acute or chronic inflammatory responses.

In addition, p38α, by other independent mechanisms, can induce the expression of metalloproteinases, which are involved in matrix formation, remodeling, or degradation by metastatic cancer cells, with the participation of the vascular endothelial growth factor A (VEGF-A), which facilitates the tumor survival through angiogenesis induction. For tumor growth, the formation of new vessels close to the cells is indispensable, a process known as angiogenesis [78]. Angiogenesis is also involved in metastasis, as it provides cancer cells with new, easy routes to spread to other parts of the body. In solid tumors, oxygen concentrations in adjacent regions are very low, a condition called hypoxia. Angiogenesis can be triggered by factors such as hypoxia, which is regulated by the p38 MAPK pathway [79,80]. Several studies using knockout mice for p38, MKK3, and MKK6 have shown that p38 is involved in the activation of hypoxia-inducible factor-1α/β (HIF-1), which in turn initiates the transcription of angiogenic factors such as VEGF [81,82,83]. As for p38β, it has been reported that it may play an important role in tumorigenesis through the regulation of lipocalin 2 (LCN2) expression, a direct target of plakophilin 3 (PKP3). Elevated LCN2 expression has been shown to lead to increased invasion, tumor formation, and metastasis in different tumor types [84]. Thus, in the absence of PKP3, p38β may control LCN2 expression, showing a potential role for p38β in tumor development [85]. In relation to angiogenesis, the direct connection of p38β with VEGF has been demonstrated in a murine model [86], suggesting the importance of this isoform in tumor vascularization and hypoxia-induced cell proliferation.

Importantly, the downstream activator of p38: MKK6 may or may not have metastatic effects, depending on the experimental model [87,88]. Finally, it has been shown that p38α, via MK2, can mediate cell migration by remodeling the actin cytoskeleton [89].

On the other hand, there is evidence that p38α may have tumor suppressor functions through genetic studies that have identified that MKK4 (a MAP2K shared by both the JNK pathway and p38) may have a relevant role in this type of process. Thus, loss-of-function alleles for MKK4 were found in some types of cancer such as lung, breast, colon, prostate, and even pancreatic cancer; however, there are also studies indicating that this loss could also promote tumorigenesis [90,91].

The tumor suppressor activity of p38α has been studied in relation to the occurrence of the overexpression of several negative modulators of p38 MAPK signaling in various human tumor types, such as the phosphatases PPM1D and DUSP26 [91,92,93] and ASK1 inhibitors [67]. Somatic mutations in the p38 MAPK pathway have also been identified in human tumors, although the correlation of these with cancer development remains unclear [94]. However, increased levels of phosphorylated p38α have also been correlated with malignancy in several types of cancer such as thyroid cancer [95]. As with p38α, p38β may also have a dual function in cancer, playing a role as a tumor suppressor, although more studies are needed to reach a firm conclusion [64].

**Figure 4:**
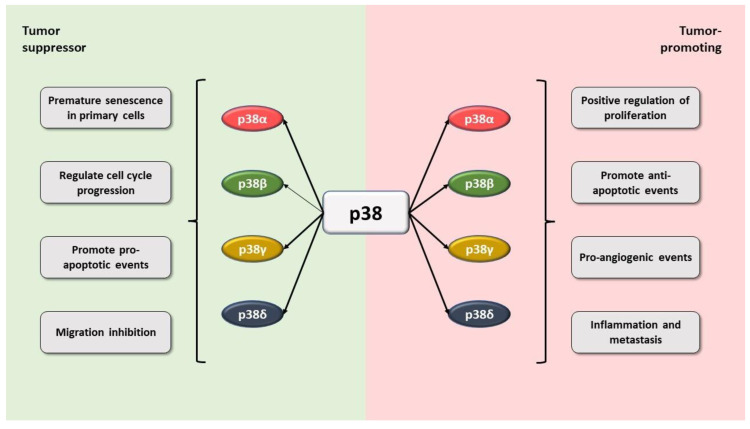
The p38 MAPK signaling pathway plays a dual role in cancer development. The p38 isoforms are able to promote both pro-oncogenic and tumor suppressive processes. Figure created using Servier Medical Art by Servier, which is licensed under a Creative Commons Attribution 3.0. Unported License: https://smart.servier.com (accessed on 23 November 2021).

By studying the expression of p38γ and p38δ in knock-out mouse models and mouse cancer models, the tumor suppressor role of these isoforms in mouse embryonic fibroblasts has been demonstrated, such that their deficiency could increase cell migration and metalloproteinase-2 secretion, thereby modifying cell contact inhibition. Furthermore, decreased expression of p38γ in K-Ras-transfected fibroblasts resulted in increased cell proliferation and tumorigenesis in trials conducted both in vitro and in vivo [96]. The pro-oncogenic role of p38γ and p38δ was confirmed in the two-step DMBA/TPA chemical skin carcinogenesis model, showing that the single deletion of p38γ or p38δ only partially blocks the development of skin tumors, whereas the totally blockade was caused by deleting both genes in combination. In addition, these isoforms have proinflammatory functions, as proinflammatory cytokine and chemokine expression in the skin was reduced and neutrophil recruitment was decreased in p38γ/p38δ-deficient mice [97]. Therefore, it can be suggested that these two isoforms would collaborate in the formation of a proinflammatory tumor microenvironment, leading to tumorigenesis. This proinflammatory role of p38γ and p38δ has been demonstrated in studies on cancer associated with colitis, with mice deficient in these isoforms, exhibiting a smaller number of tumors, correlating with less inflammation, cytokine, and chemokine production and an inflammatory cell infiltration deficiency [98] (Figure 4).

Cross signaling between pathways plays a crucial role in cell regulation, depending on the cellular context. As noted above, the JNK and p38 MAPK pathways share several upstream regulators, therefore there are many stimuli that can activate both pathways at the same time [99]. In some cases, they can synergize, activating the same transcription factors; however, in most cases, they have opposite effects, for example, in mouse models of liver cancer.

Therefore, most studies support the idea that therapeutic inhibition of the p38 MAPK pathway must depend on the cellular context, cancer type, and tumor stage, as well as the intensity and duration of the signal, together with cross signaling between MAPK pathways.

## 4. A New Approach: p38β, p38γ, and p38δ Isoforms as Cancer Biomarkers

Early detection and diagnosis of cancer is crucial for the survival of cancer patients. Tumor biomarkers are crucial in reflecting molecular differences between certain types of cancers, sometimes enabling detection at an early stage—a consequent advantage in improving a patient’s prognosis. Therefore, many studies have focused on the discovery of new biomarkers targeting the MAPK pathway and, in this sense, the p38α isoform is one of the most studied; however, in recent years, isoforms p38β, p38γ, and p38δ have been recognized with a potential role in cancer diagnosis.

The p38γ isoform is associated with invasion, clinical stage, metastasis, and chemosensitivity. Some studies have shown that overexpression of this isoform is associated with a poor prognosis in patients with cellular hepatocarcinoma [100] and may be related to the degree of malignancy of the glioma [101]. In the case of breast cancer, a correlation has been found between higher p38γ expression and a lower survival rate [102]. The p38γ isoform has also been involved in the enhancement of the epithelial–mesenchymal transition phenotype, as well as in the increase of cancer stem cells (CSCs) subpopulations [103]. In addition, it has been shown that p38γ can transduce signals that would be related with different chemotherapy drugs such as etoposide, cisplatin, and tamoxifen [104], which will be discussed in the next section. In recent years, it has become clear that certain molecules may target other components of the p38 pathway, such as the p38γ isoform. In 2020, it was found that imidazole propionate (a microbial metabolite) can reduce the efficacy of the type 2 diabetes drug called metformin by interacting with the p38γ/AKT/AMPK signaling pathway [105]. Metformin is a high-impact drug in the treatment of type 2 diabetes; however, a correlation between its use and beneficial effects in cancer therapy has been demonstrated [106]. Therefore, it has been suggested that p38γ may play an important role in microbial metabolism that affects the impact of this drug in cancer treatment [107].

It has been shown that p38δ is overexpressed in cholangiocarcinoma, invasive human cutaneous squamous cell carcinoma (SCC), head and neck SCC, and prostate cancer [108,109,110]. Regarding its functionality as a tumor suppressor, p38δ-expression knockdown has been shown to increase proliferation and tumorigenicity of cancer cells, such as glioma, melanoma, and oesophageal SCC, one of the pathways by which p38δ is silenced in melanoma and OESCC is the hypermethylation and inhibition of the transcription of its *MAPK13* gene [69,70,111,112]. Moreover, P38δ has been shown to mediate pro-oncogenic functions in colorectal cancer through its activation by MKK3 [113]. In addition, it has been suggested that both isoforms p38γ and p38δ are involved in the cardiotoxicity induced by doxorubicin treatment in breast cancer with the peculiarity that p38γ/δ systemic deletion was cardioprotective in female but not in male mice (p38δ genetic ablation protects female mice from anthracycline cardiotoxicity) [114]. Thus, the implication of p38γ and p38δ isoforms in cancer is being increasingly clarified, although their cell type-specific functions remain to be further analyzed [63]. 

The p38β is an isoform that has been recently incorporated in cancer studies and showed an interesting role in cancer development. For example, this isoform has been proposed as a key target of the Pokémon proto-oncogene, a transcription factor involved in tumorigenesis and metastasis in liver cells [115]. Furthermore, p38β has been suggested also as a potential biomarker in pancreatic cancer [116]. In hepatocellular carcinoma, p38β has been shown to be a target of miR-516a-5p, which is controlled by a novel circular RNA, circ-0001955, that increases p38β expression, facilitating hepatocellular tumorigenesis [117]. It has been also suggested that p38β overexpression could be a novel biomarker in Sezary syndrome, a variant of cutaneous T-cell lymphoma [118]. The role of p38β MAPK has also been studied as a target for therapy against cancer-induced muscle atrophy or cachexia, as it acts on the phosphorylation of p300, which mediates muscle wasting [119]. In addition, the downregulation of *MAPK11* gene expression in specific female tissue cancers has been determined with in-silico studies [120]. Finally, it is noteworthy that, in the specific subset of lung cancer in non-smokers in China, p38β was found to be overexpressed [121].

## 5. p38 as a Pharmacological Target in Cancer

The p38 pathway is a pleiotropic cascade with different actions in the proper functioning or otherwise of cells in the body, such as in response to growth factors or stress, during carcinogenesis or metastasis. For this reason, p38 plays a fundamental role in the approach to conventional cancer therapies, such as radiotherapy or chemotherapy and targeted treatments, using certain small molecules that act as inhibitors of key steps in the signaling pathway, although they can induce resistance from the first dose due to the positive selection of those tumor cells that are able to compensate the specific target pathway.

### 5.1. Impact of p38 in Radiotherapy

A large percentage of patients are treated with ionizing radiation for cancer. The effect of exposure to this radiation on both normal and tumor tissues has been extensively studied in recent years. The early effects of chemotherapy result in the induction of apoptotic cell activation, with excessive secretion of proinflammatory cytokines because of this process, as well as the increased recruitment of blood cells and platelets, the activation of coagulation systems, and increased vascular permeability [122]. The molecular signaling mechanism induced by radiotherapy remains unclear, but, in recent years, the involvement of the p38 MAPK pathway has been described in some studies.

For example, it has been shown that, in human dermal microvascular endothelial cells, radiation-induced apoptosis is largely mediated by p38 [123] and this pathway could be inhibited by the overexpression of Bcl2. In addition, p38 may contribute through its promotion of cell cycle arrest by the p53/p21/p16 axis and regulation of proinflammatory cytokine production and may be considered to contribute to cellular senescence [124].

### 5.2. Involvement of p38 in Chemotherapy

Apoptosis is a mechanism of cell death in response to anticancer agents and, as mentioned before, p38 plays a key role in this process, modulating the chemotherapeutic response to both conventional and targeted therapies. P38 has been implicated in cell apoptosis that is mediated by cis-platinum and 5-fluorouracil (5-FU) in breast and colon cancer cell lines [125] and has also been associated with resistance to gemcitabine and cytarabine [125,126]. In this way, it has been shown how p38β could be a major determinant of gemcitabine’s radiosensitivity potential without involving the frequent p38α isoform, suggesting that the role of the other p38 isoforms should be further explored [127].

P38 contributes to chemotherapeutic resistance in several tumor types, e.g., in response to doxorubicin, cis-platinum, or other agents that target DNA damage, as p38 will promote cell survival of p53-deficient cancer cells through MK2-mediated cell cycle arrest [128,129]. In fact, the inhibition of p38 in mouse models of breast and colon tumors has enhanced the effects of conventional chemotherapies such as cis-platinum, 5-FU, or irinotecan in several studies [130]. 

Antiestrogen tamoxifen is a key treatment for hormone-sensitive breast cancers, and the activation of the p38 pathway is related to resistance and poor prognosis [131]. However, the inhibition of VEGF activity via the p38 pathway could alleviate tamoxifen resistance [132], as p38 activity was elevated in xenografts of breast cancer cells that were resistant to tamoxifen [133]. 

In terms of p38γ-related drugs, it has been shown that this isoform can regulate the signal transduction of drugs targeting DNA topoisomerase II α, which represent an important class of antitumor drugs in the clinic, thus enhancing their therapeutic activity [134]. 

However, it has been shown that the role of the p38 pathway in the response to chemotherapy depends on the type of tumor, since each tumor microenvironment also participates in the neoplasia. The involvement of this pathway in tumor development involves several cell types within the tumor microenvironment, as discussed before. Thus, several p38 inhibitors have been shown to inhibit cancer progression by halting processes such as angiogenesis, for example, in an in vivo experimental model of prostate cancer [135], as well as in squamous cell carcinoma of the head and neck [136]. It is therefore not uncommon to think that different inhibitors will be developed that specifically target p38 to inhibit processes such as angiogenesis and metastasis. 

In addition, genotoxic, ROS-generating drugs such as doxorubicin induce the liberation of IL-6 from endothelial cells in a mechanism that depend on p38 activation, contributing to the induction of senescence in tumor-associated stromal cells [137,138]. The senescent stromal cell inflammatory secretome (SASP) is a microenvironmental factor that promotes p38-regulated tumor progression [139] such that the inhibition of p38 may compromise the protumorigenic capabilities of the microenvironment. 

All these studies demonstrate the viability of therapy directed against the p38 pathway as a target for cancer, and are summarized in the following section. 

### 5.3. Drugs with Pharmacological Potential Targeting the p38 MAPK Pathway

There are many clinical trials evaluating the role of p38 as a biomarker and target for different diseases, with a considerable percentage dedicated to cancer treatment, where p38 is considered a promising target, either using p38-specific inhibitors alone or in combination with other chemotherapeutic agents [125] (Table 1).

However, one cannot overlook the fact that p38 is involved in many biological activities, and its inhibition could lead to unwanted and unknown side effects, therefore efforts are currently focused on discovering agents that specifically target p38 or a component of the pathway that is deregulated and is the trigger for a particular disease. For example, small peptides are being developed that target VEGFR-2, thereby inhibiting the activation of p38 by VEGF, and thus inhibiting p38-dependent angiogenic effects [14]. 

In this regard, it has also been shown that the combination of p38 inhibitors with conventional chemotherapy drugs could be a promising approach in antitumor therapy. One of the most prominent is the p38α and p38β inhibitor LY2228820/Ralimetinib, which has antiangiogenic effects in vitro and in vivo [150] and has been tested in a phase I study in combination with tamoxifen in patients with advanced cancer [151]. This treatment showed safety, tolerability, and an appropriate pharmacokinetics for women with advanced breast cancer and 21.3% of patients achieved stable disease without progression. A trial combining Ralimetinib with gemcitabine and carboplatin is currently underway in a phase II trial in women with platinum-sensitive ovarian cancer. It is also being tested both alone and in combination with other agents such as radiotherapy and temozolomide for glioblastoma [152,153].

Because of the involvement of p38α in proinflammatory cytokine production and cytokine receptor signaling, the development of a large number of small p38α MAPK inhibitor molecules is ongoing (Table 1). These have shown high specificity towards p38α in vitro; however, few have gone beyond phase I/II clinical trials due to liver toxicity [76]. In this regard, we highlight the trial with talmapimod (SCIO-469) in multiple myeloma [154]. However, we must consider the role of p38α as a tumor suppressor [155,156] and whether the inhibition of p38α could result in an increased predisposition to cancer, as supported by experiments in mouse models of lung and liver cancer [66,157]. As for the p38β isoform, it has been shown that peptide inhibitors for this isoform are able to induce toxicity in pancreatic cancer cell lines such as PANC-1, which could be a potential therapeutic implication [85], but, unfortunately, we have found no other inhibitor assays that specifically target this or other p38 isoforms apart from p38α, since, due to their sequence homology, the vast majority of p38α inhibitors also inhibit p38β. BIRB796 is a very potent p38α/p38β inhibitor that also blocks p38γ and p38δ, albeit at higher concentrations [158]. However, several analogues with greater potential and specificity targeting p38δ have been developed [159], though could still inhibit p38α. Selective inhibitors have been developed for p38γ and p38δ in vitro and in cultured cells, which do not inhibit p38α through their differences in the residues they possess at the hinge ATP-binding pocket [160]; however, this specificity needs to be studied further.

Moreover, treatment resistance should be considered in cancer therapy, and attempts should be made to limit it. Thus, the inhibition of the p38 pathway has been shown to limit resistance to irinotecan in colon adenocarcinoma [161]. However, it is not possible to predict which tumors would respond well to combination therapies. Therefore, predictive biomarkers would be useful to select those patients who could benefit from these therapies. In this sense, MK2 inhibition has been proposed as an alternative to reduce unwanted effects. MK2 (a p38-regulated kinase involved in several processes such as apoptosis, cell cycle, and oxidant-induced stress response) induces the G2/M checkpoint response, thus contributing to tumor growth and the invasiveness of certain types of cancer [17]. Thus, MK2-deficient mice are unlikely to develop skin tumors due to low proinflammatory activity and increased apoptosis [162], making MK2 a potential drug target. Moreover, it has been shown that the inhibition of MK2 could lead to the regression of p53-deficient tumors in vivo, following treatment of mice with cisplatin and doxorubicin [128]. Finally, in combination with the inhibitor of apoptosis proteins (IAP)’s inhibitors, acute myeloid leukemia cells could be targeted [163].

Almost all clinical trials to date have failed and therefore no compound has yet been approved, which has been due to systemic side effects on the heart, liver, or nervous system [61], which could be attributed to the large number of substrates on which MAPK acts [59]. Because of this, there is a need for more tailored approaches, targeting a specific cell type or a particular p38-driven signaling pathway, which may be more successful than generalized inhibition. In addition to targeting p38 signaling, compounds that induce p38 or degradation together with the possibility of tissue-specific targeting have been considered [164,165]. Moreover, the inhibition of the nuclear translocation of p38α, which is involved in p38α functions in the nucleus, has been shown to help to reduce inflammation in mouse models [166].

Therefore, efforts should focus on gaining a better understanding of the contribution of p38 to the development of cancer and other diseases, both at the cellular and organismal level, to develop better compounds that specifically target the different components of this MAPK signaling pathway to improve responses to treatment and reduce the drawbacks of conventional inhibitors.

## 6. Conclusions and Future Perspectives

MAP kinases are involved in the response of cancer cells to a wide range of stimuli, both external and internal. In the last 25 years, many important advances have been made in our understanding of the regulation and functionality of the p38 MAPK pathway. Due to genomic and proteomic strategies, the different components of the p38 MAPK pathway have been identified that could play a key role in oncogenic processes when they are altered, making them potential targets for the development of antitumor drugs. However, these strategies have been hampered by the activation of compensatory pathways in cancer cells and the high degree of crossover and dual functionality that exists between the other different MAPK families (ERK and JNK), thus giving rise to a strong resistance to the most common treatments. These kinases are highly regulated at various levels, where numerous miRNAs have been described as interfering in different steps of signaling. However, numerous reports have shown that the use of miRNA as biomarkers or drug targets has been unsuccessful due to differences in the methodology of the studies, the lack of standard methods for normalization, the different miRNA processing, and the inability to discriminate among closely related miRNAs, among others.

Given the latest advances in gene therapy, it is not impossible to think that cancer, which can be defined as a disease caused by different genetic predispositions, could be fought by modifying certain aberrations in the genome or by inducing certain tumor suppressor genes, a revolutionary process also known as epigenome editing. This would be possible thanks to the CRISPR-Cas9 method that could inhibit oncogenes such as the p38γ gene in some tumors, which could be a promising cancer therapeutic strategy [145]. Several studies have shown that CRISPR-Cas9 could inhibit in vitro cancer cell progression, proliferation, and migration in human colorectal cancer by inducing apoptosis [167].

It should be noted that immunotherapy has become an important cancer treatment in recent years. These include immunosuppressants that target PD-1 on T cells and PD-L1 on tumor cells. The aim of this antitumor therapy is to activate CD8+ T cells so that they can kill tumor cells specifically. However, when this occurs, there are immune checkpoints aimed at preventing these processes, resulting in immunosuppression and consequent immune escape from tumors [168]. It has been suggested that the inhibition of p38 may facilitate the positive selection of CD4+ and CD8+ cells, so that the use of p38-targeted inhibitors combined with T-cell immunotherapy could develop effective antitumor approaches [169]. 

However, a better understanding of the contribution of p38 to disease at both the cellular and organismal levels is needed and can be achieved by better defining the specific functions of this signaling pathway in specific tumors. In addition, future therapies may also involve collateral cancer processes such as directly related as angiogenesis or the tumor microenvironment modulation. 

Moreover, the existence of several isoforms that have been located at different levels of expression depending on the tissues and the degree of tumorigenicity opens the door to explore new diagnostic strategies based on the different expression and/or activity of these peculiar kinases. Recent studies have shown that the differentiation and quantification of individual alternative isoforms could improve insights into cancer diagnosis and outcome. However, there are limitations in the traditional methods used for determining isoforms, such as the level of transcription, low level of expression, and cross-linking of activity.

Nevertheless, there is no doubt that, in the coming years, the development of new drugs targeting p38 will be able to improve therapeutic efficacy in diseases such as cancer in a successful way, in view of favoring a medicine of greater precision.

## Figures and Tables

**Table 1:** Preclinical and clinical trials in recent years aimed at testing inhibitors targeting components of the p38 MAPK pathway as cancer treatments.

Treatment	Clinical/Preclinical Trial Title	Study Features	Clinicaltrials.gov Identifier/Reference
Ralimetinib (LY2228820) + Carboplatin + Gemcitabine	A study LY2228820 for recurrent ovarian cancer	Clinical trial Phase Ib/II Targeting p38α and p38β isoforms by combining the action of carboplatin radiotherapy with carboplatin and gemcitabine chemotherapy	NCT01663857
Ralimetinib + Tamoxifen	A multicenter trial assessing the efficacy and safety of Tamoxifen Plus LY2228820 in advanced or metastatic breast cancer progressing on aromatase inhibitors (OLYMPE)	Clinical trial Phase II Targeting p38α and p38β isoforms by combining the action of tamoxifen therapy, a selective estrogen receptor modulator	NCT02322853
Ralimetinib + Midazolam + Tamoxifen	A study of LY2228820 in participants with advanced cancer	Clinical trial Phase I Targeting p38α and p38β isoforms by combining the action of tamoxifen therapy, a selective estrogen receptor modulator with midazolam, to promote the pharmacological action of compounds	NCT01393990
Ralimetinib + Temolozomide (TMZ) + Radiotherapy	Study of LY2228820 with radiotherapy Plus concomitant TMZ in the treatment of newly diagnosed glioblastoma (GLYRad)	Clinical trial Phase I/II Targeting p38α and p38β isoforms by combining the action of TMZ, aimed at inhibiting cell proliferation	NCT02364206
Prexasertib ^1^ (LY2606368) + Ralimetinib	A study of Prexasertib (LY2606368) in combination with Ralimetinib in participants with advanced or metastatic cancer	Clinical trial Phase I Targeting p38α and p38β isoforms by combining the action of a checkpoint kinase inhibitor	NCT02860780
Talmapimod (SCIO-469)	Open-label study for patients with myelodysplastic syndromes	Clinical trial Phase II Targeting selectively p38α isoform, although it also shows selectivity on p38β and other MAPKs	NCT00113893
rCisplatin + PH-797804	Inhibition of p38 MAPK sensitizes tumor cells to cisplatin-induced apoptosis mediated by reactive oxygen species and JNK	Murine model with induced mammary tumors. p38 inhibition enhances cisplatin cytotoxicity	[140]
CDD-111 and CDD-450	Inhibition of the stromal p38MAPK/MK2 pathway limits breast cancer metastases and chemotherapy-induced bone loss	Murine model implanted with cancer cells. p38 inhibition enhances cisplatin cytotoxicity	[141]
Sorafenib and BIRB796, L-skepinone or PH-797804	In vivo RNAi screening identifies a mechanism of sorafenib resistance in liver cancer	Murine model with NRAS^G12V^ and p19*Arf*-knockout liver tumors. p38 inhibition increases therapeutic efficacy of sorafenib	[142]
PH-797804	Dual function of p38α MAPK in colon cancer: suppression of colitis-associated tumor initiation but requirement for cancer cell survival	Murine model with AOM/DSS inflammation-driven colon tumors. p38 inhibition reduces colon tumor load	[143]
PF3644022 + PF477736	A synergistic interaction between Chk1- and MK2 inhibitors in KRAS-mutant cancer	Murine model with KRAS^G12D^ and *Tp53*-knockout lung tumors, high-grade sarcomas or BRAF-driven intestinal carcinomas. Combined inhibition of MK2 and CHK1 induces cytostatic or cytotoxic effects in different tumor types	[144]
BIRB796	Multi-phenotype CRISPR-Cas9 screen identifies p38 kinase as a target for adoptive immunotherapies	Mice with subcutaneously implanted melanoma cell line B16-mhgp100 or injected with the acute lymphoblastic leukemia cell line E2a-PBX. p38 inhibition in T cells ex vivo increases their immunosuppression properties in vivo	[145]
LY2228820	Blockade of p38 kinase impedes the mobilization of protumorigenic myeloid populations to impact breast cancer metastasis	Mice with mammary tumors formed by implantation of the mouse mammary carcinoma cell line 4T1. p38 inhibition reduces tumor growth and recruitment of protumoral myeloid cells	[146]

^1^ Prexasertib (LY2606368) is a novel checkpoint kinase inhibitor (CHK) under investigation for the treatment of various types of cancer [147,148,149].

## Data Availability

Not applicable.
